# Supplementary material for: Fibrodysplasia ossificans progressiva mutant ACVR1 signals by multiple modalities in the developing zebrafish
Source: eLife. 2020 Sep 8;9:e53761. doi: 10.7554/eLife.53761 (PMC7478894; doi:10.7554/eLife.53761)
Supplement: Transparent reporting form [file elife-53761-transrepform.docx]

***eLife’s* transparent reporting form**

We encourage authors to provide detailed information *within their submission* to facilitate the interpretation and replication of experiments. Authors can upload supporting documentation to indicate the use of appropriate reporting guidelines for health-related research (see [EQUATOR Network](http://www.equator-network.org/%20)), life science research (see the [BioSharing Information Resource](https://biosharing.org/)), or the [ARRIVE guidelines](http://www.plosbiology.org/article/info:doi/10.1371/journal.pbio.1000412) for reporting work involving animal research. Where applicable, authors should refer to any relevant reporting standards documents in this form.

If you have any questions, please consult our Journal Policies and/or contact us: [editorial@elifesciences.org](mailto:editorial@elifesciences.org).

**Sample-size estimation**

- You should state whether an appropriate sample size was computed when the study was being designed
- You should state the statistical method of sample size computation and any required assumptions
- If no explicit power analysis was used, you should describe how you decided what sample (replicate) size (number) to use

Please outline where this information can be found within the submission (e.g., sections or figure legends), or explain why this information doesn’t apply to your submission:

Replicates: All experiments were replicated at least twice. Different aliquots of mRNA or morpholino were used for each replicate to control for differences in aliquots.

Sample number: Within each replicate all mRNAs were injected through the same calibrated needle into embryos within different conditions to ensure consistency in mRNA injection. For each condition within each replicate approximately 60 embryos were injected. 60 embryos is the maximum number of embryos that can be held together in a single petri dish. Once unfertilized and dead embryos are removed at 4 hours post fertilization, this typically left at least 40 embryos for analysis.

Because the phenotypes occur on an 11 point scale usually 20 embryos is sufficient to determine the range of phenotypes in a single experiment.

In experiments where no genotyping was necessary, 30 total embryos from at least 2 experiments was sufficient to observe consistent rescue from C5 phenotypes (ie fig 1). 50 embryos or more was preferred for observing changes in rescue intensity (ie ligand response experiments; fig 3 and S3).

In cases where genotyping following analysis was necessary, typically 25% of analyzed embryos would be within the homozygous mutant category. 20 embryos was sufficient to observe rescue from C5 phenotypes (ie fig 4 and 5). While for ligand response experiments in which ranges of phenotypes were observed (fig 6) we aimed to have at least 30 embryos in each category.

Due to the reproducibility of the pSmad1/5 immunostains and dorsal marker *in situs*, a minimum of 3 embryos from 2 different experiments was sufficient to show rescue or enhancement of signaling.

**Replicates**

- You should report how often each experiment was performed
- You should include a definition of biological versus technical replication
- The data obtained should be provided and sufficient information should be provided to indicate the number of independent biological and/or technical replicates
- If you encountered any outliers, you should describe how these were handled
- Criteria for exclusion/inclusion of data should be clearly stated
- High-throughput sequence data should be uploaded before submission, with a private link for reviewers provided (these are available from both GEO and ArrayExpress)

Please outline where this information can be found within the submission (e.g., sections or figure legends), or explain why this information doesn’t apply to your submission:

Experimental replicates are reported within the figure legends

Biological replicate: each injected embryo within a condition (referred to as N values for each column in the figure legends; total Ns from combined technical replicates are reported)

Technical replicate: each separate injection (referred to as “experiment” in reported “pooled experiments” within the figure text)

Technical replicates were excluded if the associated control injections performed in the same experiment did not produce the expected results. (ie the *acvrl1* morpholino failed to generate a C4-5 dorsalized phenotype or *bmp7* mRNA failed to rescue *bmp7-/-* fish)

Within technical replicates all outliers were included in data with the exception of embryos that displayed significant cell death, presumably from morpholino toxicity. Morpholino toxicity can be identified by significant cell death within the neural tissue of the embryo and creates a dark coloration. This cell death confounds ventralized phenotypes which have reduced eyes and small heads.

A pSmad1/5 immunostaining experiment was excluded from the results due to unexpected non-specific staining that was not observed in other experiments.

For quantitative pSmad1/5 and pSmad2 fluorescence measurements, the mean fluorescence was calculated within each nuclei of the embryo. Nuclei with mean fluorescence outside 1.8 times the interquartile range were excluded to determine the final mean fluorescence of the whole embryo. All individual measured embryos were kept for analysis. No embryos were excluded.

For quantitative HA fluorescence, embryos that fell outside 1.8 times the interquartile range were excluded. (0-1 embryos per condition)

No high-throughput sequence data was generated in this study

**Statistical reporting**

- Statistical analysis methods should be described and justified
- Raw data should be presented in figures whenever informative to do so (typically when N per group is less than 10)
- For each experiment, you should identify the statistical tests used, exact values of N, definitions of center, methods of multiple test correction, and dispersion and precision measures (e.g., mean, median, SD, SEM, confidence intervals; and, for the major substantive results, a measure of effect size (e.g., Pearson's r, Cohen's d)
- Report exact p-values wherever possible alongside the summary statistics and 95% confidence intervals. These should be reported for all key questions and not only when the p-value is less than 0.05.

Please outline where this information can be found within the submission (e.g., sections or figure legends), or explain why this information doesn’t apply to your submission:

Most data was analyzed for the ability to rescue severely dorsalized C5 phenotypes to less dorsalized or ventralized phenotypes (by phenotyping, pSmad1/5 immunostain or in situ)

Experiments involving enhancement of phenotype were analyzed the ability to shift the majority of observed phenotypes from one general category of phenotypes to another (more general categories being: severely dorsalized (C5 and C4), mildly dorsalized (C3-C1), wildtype, mildly ventralized (V1-V3) and severely ventralized (V4 and V5)). Alternatively, we assessed enhanced ventralization based on change in the most ventralized phenotype observed in the condition.

In the case of the *in situs* in Fig 6 the ability to shift embryos from a C5 dorsalized phenotype, to a less dorsalized phenotype or ventralized phenotypes.

All quantitative immunofluorescence data was compared using a two-tailed student’s t-test assuming unequal variances. Data was deemed statistically significant if P<0.05.

(For large datasets, or papers with a very large number of statistical tests, you may upload a single table file with tests, Ns, etc., with reference to sections in the manuscript.)

**Group allocation**

- Indicate how samples were allocated into experimental groups (in the case of clinical studies, please specify allocation to treatment method); if randomization was used, please also state if restricted randomization was applied
- Indicate if masking was used during group allocation, data collection and/or data analysis

Please outline where this information can be found within the submission (e.g., sections or figure legends), or explain why this information doesn’t apply to your submission:

Experimental groups were determined based on genotype and injection conditions. This information can be found within the figure and figure legends for all tables.

Phenotypic analysis of bmpr1a mutant embryos was carried out blind followed by genotyping.

**Additional data files (“source data”)**

- We encourage you to upload relevant additional data files, such as numerical data that are represented as a graph in a figure, or as a summary table
- Where provided, these should be in the most useful format, and they can be uploaded as “Source data” files linked to a main figure or table
- Include model definition files including the full list of parameters used
- Include code used for data analysis (e.g., R, MatLab)
- Avoid stating that data files are “available upon request”

Please indicate the figures or tables for which source data files have been provided:

Included are excel files containing total numbers of embryos within each phenotypic category in each condition of reported tables. These numbers were converted into percent of total embryos to generate the bubble graphs using the excel bubble graph series template. Within the excel sheet, each tab shows data for a separate table.

Included are also, an excel sheet with the raw nuclear fluorescence for all pSmad1/5 and pSmad2 immunostained embryos, and the mean nuclear fluorescence of each embryo before and after nuclei outside the 1.8x IQR were excluded. A second excel sheet shows the comparison of the means within each group and the statistical tests performed between them.

A fourth excel sheet shows data for the quantitative HA stain. Surface area of βcatenin was measured and the HA fluorescence within that surface area was measured, then the HA fluorescence/um βcatenin was calculated. A separate tab shows the exclusion of embryos that fell outside 1.8x the IQR and the statistical tests performed between groups.
